# Gastrointestinal Cancers and Liver Cirrhosis: Implications on Treatments and Prognosis

**DOI:** 10.3389/fonc.2021.766069

**Published:** 2021-10-21

**Authors:** Ze Xiang, Yiqi Li, Chaojie Zhu, Tu Hong, Xianglin He, Hua Zhu, Danbin Jiang

**Affiliations:** ^1^ Department of Gastroenterology, Yancheng Third People’s Hospital, Yancheng, China; ^2^ School of Medicine, Zhejiang University, Hangzhou, China; ^3^ Chu Kochen Honors College, Zhejiang University, Hangzhou, China; ^4^ Department of Hepatobiliary and Pancreatic Surgery, The First Affiliated Hospital, School of Medicine, Zhejiang University, Hangzhou, China

**Keywords:** gastrointestinal cancer, liver cirrhosis, postoperative complications, cancer treatment, prognosis

## Abstract

Liver cirrhosis tends to increase the risk in the management of gastrointestinal tumors. Patients with gastrointestinal cancers and liver cirrhosis often have serious postoperative complications and poor prognosis after surgery. Multiple studies have shown that the stage of gastrointestinal cancers and the grade of cirrhosis can influence surgical options and postoperative complications. The higher the stage of cancer and the poorer the degree of cirrhosis, the less the surgical options and the higher the risk of postoperative complications. Therefore, in the treatment of patients with gastrointestinal cancer and liver cirrhosis, clinicians should comprehensively consider the cancer stage, cirrhosis grade, and possible postoperative complications. This review summarizes the treatment methods of patients with different gastrointestinal cancer complicated with liver cirrhosis.

## Introduction

Gastrointestinal cancers and liver cirrhosis are common diseases worldwide. A significant number of patients with gastrointestinal cancer also suffer from liver cirrhosis. Gastrointestinal tumors mainly include esophageal cancer, liver cancer, gastric cancer, pancreatic cancer, and colorectal cancer (1, 2). Gastrointestinal cancer is the third most common cause of cancer-related death worldwide, and its incidence is on the rise globally. More than 15% of newly diagnosed cancer cases and 17% of cancer deaths are associated with gastrointestinal cancers (3, 4). The treatment of gastrointestinal cancers has been a focus of research due to their high morbidity and mortality (5). As a common complication of gastrointestinal cancer, liver cirrhosis makes its treatment more complicated.

It is well known that liver cirrhosis is not only a risk factor of primary liver cancer but also increases the risk of extrahepatic malignancies. Compared with non-cirrhotic patients, patients with cirrhosis had an increased risk of poor prognosis for non-hepatic abdominal surgery (6). More and more studies have confirmed that the presence of cirrhosis has a great impact on the surgical outcome and prognosis of patients with gastrointestinal cancers. In this review, we summarize the treatment for patients with different gastrointestinal cancers complicated with liver cirrhosis and focus on the analysis on the influence of different grades of liver cirrhosis on the treatment and postoperative complications, aiming to provide guidance for treatment options for patients with gastrointestinal cancers and liver cirrhosis.

## General Introduction of Liver Cirrhosis

Liver cirrhosis (LC) is the final stage of liver fibrosis and a wound-healing reaction of chronic liver injury. The main causes of liver cirrhosis include alcoholic hepatitis, hepatitis B, hepatitis C, and non-alcoholic fatty hepatitis (7). LC is characterized by abnormal liver structure and function, accompanied by fibrous septum and nodule formation and changes in blood flow (8). The process of LC has two stages. The LC compensated period is a long-term asymptomatic phase of fibrosis, while LC decompensated period is a rapidly progressive phase with complications of portal hypertension and liver function impairment, including ascites, varicose vein bleeding, encephalopathy, jaundice, and more (9–11). LC is usually accompanied by complex alterations in the hemostatic system. Patients suffering from LC have few platelets and prolonged prothrombin time, resulting in a high rate of bleeding during surgery (12, 13). Therefore, the presence of liver cirrhosis increases the risk of treatment for gastrointestinal cancers.

Prognostic models and staging systems are instructive for the appropriate treatment of patients with liver diseases (14–16). Patients with gastrointestinal cancer and cirrhosis were mainly involved in two broad staging systems, namely, the Child–Turcotte–Pugh (CTP) score and Model for End-Stage Liver Disease (MELD) score.

The CTP score was first proposed by Child and Turcotte to predict the outcome of patients receiving portal shunt for variceal bleeding. The grades of encephalopathy and ascites and serum bilirubin, albumin, and prothrombin time were integrated into the scoring model for comprehensive consideration (17). It is also believed that the prothrombin index or international normalized ratio (INR) can be used instead of prothrombin time (14). Each variable is assigned 1–3 points. According to the specific conditions of LC, patients can be divided into three prognostic subgroups: CTP A grade (5–6 points), B grade (7–9 points), and C grade (10–15 points) (14, 17). The main limitations of the CTP score are the cutoff value of different variables and the clinical variables that need to be included in the subjective assessment (14).

The MELD score was originally designed to predict the prognosis of transjugular intrahepatic portal system shunt and was later found to be an accurate predictor of mortality in patients with end-stage liver diseases (18). The three objective variables of serum bilirubin, serum creatinine, and INR were integrated into the MELD scoring model. Compared with the CTP score, the main advantage of the MELD score model is that it is more finely layered. In addition, it includes serum creatinine, which is an important factor predicting the survival of patients with liver disease (19–21). Its limitations include the need for calculation, which makes it less convenient than the CTP score to be used in daily clinical practice and the lack of well-defined subcategories to assess the risk of personal death (14).

The staging of cirrhosis plays an important role in the treatment of patients with gastrointestinal cancers and cirrhosis. Patients with mild cirrhosis can receive most cancer treatments without serious postoperative complications, while patients with severe cirrhosis can only be treated conservatively.

## Treatment of Gastric Cancer With Cirrhosis and Its Influencing Factors

The presence of LC has a great influence on the surgical effect and prognosis of patients with gastric cancer. In a central cohort study, Zhou et al. explored the independent risk factors of gastrectomy postoperative complications in patients with gastric cancer and cirrhosis, and they found that cirrhosis is the largest independent risk factor for postoperative complications (22). This is consistent with the conclusion drawn by Jeong et al. (23). Similarly, Zullo et al. confirmed that cirrhosis appears to be a risk factor for the development of gastric cancer (24). The existence of LC increases the difficulty in the treatment of gastric cancer patients. The cirrhosis grade, gastric cancer stage, and the choice of different treatments have been discussed in several studies (**Table 1**). So far, a variety of treatment modalities have been reported for the treatment of gastric cancer, including endoscopic mucosal resection (EMR), endoscopic submucosal dissection (ESD), gastrectomy, lymph node dissection, and other treatment methods.

**Table 1:** Summary of gastric cancer stage, cirrhosis grade, surgical choice and prognosis.

Authors	Cancer stage	Cirrhosis grade	Management	Prognosis
**Chang et al. (** 23)	EGC	Unknown (n = 1)	EMR	The patient had good condition after 4 years’ follow-up.
**Horiki et al. (** 24 **)**	EGC	Unknown (n = 4)	EMR	No patients died of gastric cancer during the 10-year follow-up.
**Kato et al. (** 25 **)**	EGC	CTP A (n = 53)CTP B (n = 16)CTP C (n = 1)	ESD	Patients with CTP A cirrhosis had good prognosis while patients with CTP B and C benefited less.
**Choe et al. (** 26 **)**	EGC	CTP A (n = 32)CTP B (n = 11)	ESD	The recurrence rate of gastric cancer was low and recurrent gastric cancer can be resected with additional ESD.
**Barakat et al. (** 27 **)**	EGC	CTP A (n = 86)CTP B (n = 25)CTP C (n = 1)	ESD	There was no mortality related to the ESD procedure.
**Watanabe et al. (** 28 **)**	EGC	CTP A (n = 1)	ESD	No complications were observed after ESD.
**Ogura et al. (** 29 **)**	EGC	CTP A (n = 9)CTP B (n = 6)	ESD	Three patients underwent additional ESD due to recurrence.
**Ikeda et al. (** 30 **)**	EGCAGC	CTP A (n = 15)CTP B (n = 10)	Gastrectomy	Compared with CTP A patients, CTP B patients were more likely to have postoperative intractable ascites.
**Kang et al. (** 31 **)**	EGCAGC	CTP A (n = 41)	Gastrectomy	Postoperative complications were observed in 22 patients.
**Kim et al. (** 32 **)**	EGCAGC	CTP A (n = 62)CTP B (n = 12)CTP C (n = 1)	LGOG	The postoperative mortality rate was 1.3%. The overall prognosis of LG is better than that of OG.
**Alshahrani et al. (** 33 **)**	EGC	CTP A (n = 67)CTP B (n = 8)	LGOG	The overall prognosis of LG is better than that of OG.
**Zhu et al (** 34 **)**	EGCAGC	CTP A (n = 17)CTP B (n = 4)CTP C (n = 1)	LGOG	The overall prognosis of LG is better than that of OG.
**Iwabu et al (** 35 **)**	AGC	CTP B (n = 1)	Gastrectomy with D1 LN dissection	The patient had no severe complication.
**Ryu et al. (** 36 **)**	EGCAGC	CTP A (n = 26)	Gastrectomy with D2 LN dissection	There was no postoperative surgical morbidity and mortality.
**Lee et al. (** 37 **)**	EGCAGC	CTP A (n = 84)CTP B/C (n = 12)	Gastrectomy with D2 LN dissection	Two patients who had prophylactic intra-operative drains died after the operation.
**Jang et al. (** 38 **)**	EGCAGC	CTP A (n = 46)CTP B/C (n = 11)	Gastrectomy with LN dissection	22 patients developed postoperative complications and 5 patients died.
**Guo et al. (** 39 **)**	EGCAGC	CTP A (n = 25)CTP B (n = 31)CTP C (n = 2)	Gastrectomy with LN dissection	34 patients had postoperative severe complications.
**Nishimura et al. (** 40 **)**	EGC	CTP C (n = 1)	Gastrectomy after liver transplantation	The patient’s postoperative course was uneventful.
**Takechi et al. (** 41 **)**	AGC	CTP B (n = 1)	LECS	There were no short-term complications and no recurrence during 6-month follow-up.

EGC, Early gastric cancer; AGC, advanced gastric cancer; CTP A/B/C, Child–Turcotte–Pugh score A/B/C; EMR, endoscopic mucosal resection; ESD, endoscopic submucosal dissection; LG, laparoscopic gastrectomy; OG, open gastrectomy; LN, lymph node; LECS, Laparoscopic and endoscopic cooperative surgery.

### EMR and ESD

EMR and ESD are commonly used for the treatment of early gastric cancer (EGC). EMR is suitable for lesions smaller than 10–15 mm with a very low probability of advanced histology (25). Chang et al. reported a 58-year-old male patient with gastric cancer and cirrhosis who had a good prognosis after receiving EMR. Although focal submucosal invasion occurred, there was no evidence of gastric cancer recurrence (26). In addition, Horiki et al. reported in a retrospective cohort study that four patients with gastric cancer and cirrhosis received EMR, with no patients died of the cancer and no postoperative complications occurred in the 10-year follow-up period. Therefore, it is believed that EMR is safe for patients with gastric cancer and severe comorbidities (27).

ESD enables the overall excision of large or ulcerative lesions based on EMR. A multicenter retrospective study from Japan showed that patients with CTP A cirrhosis and no hepatic cell carcinoma history are good candidates for ESD, while CTP B or C patients or with histories of HCC would benefit less from receiving ESD. In the study, LC patients had complications of bleeding and perforation (28). Choe et al. mentioned that ESD could be employed in patients with EGC and LC and in CTP B cirrhosis patients. Postoperative complications, such as bleeding and perforation, may occur (29). Barakat et al. evaluated the safety and effectiveness of ESD, and they confirmed that it is safe for EGC patients with LC to receive ESD, and ESD can effectively control the bleeding caused by CTP B or C cirrhosis during surgery (42). In addition, many studies have also proved that ESD is effective and can achieve a high overall resection rate (30, 31).

According to the above studies, EMR and ESD are suitable for EGC, and the main postoperative complications are bleeding and perforation. ESD is safe and feasible for patients with gastric cancer and CTP A cirrhosis. Due to the increased risk of bleeding, there are different opinions on whether patients with CTP B and C cirrhosis should receive ESD. More studies are needed to explore whether patients with CTP B and C cirrhosis would be beneficial to receive ESD.

### Gastrectomy

Radical gastrectomy is indicated for patients with stage IB–III gastric cancer (32). Ikeda et al. compared the prognosis of gastric cancer patients with CTP A cirrhosis and those with CTP B cirrhosis after gastrectomy. They found that radical gastrectomy is safe and feasible for patients with CTP A and CTP B cirrhosis, and the most common postoperative complication is refractory ascites (33).

Radical gastrectomy mainly includes laparoscopic gastrectomy (LG) and open gastrectomy (OG). Kang et al. confirmed that LG is a feasible surgical method for patients with moderate liver dysfunction (34). Kim et al. found that patients had complications of ascites, gastric stasis, and wound infection after radical gastrectomy. After comparing the surgical outcomes of LG and OG in patients, they determined that LG combined with lymph node (LN) dissection is safer than OG in gastric cancer patients with CTP A and B cirrhosis (35). In addition, a retrospective study from Korea concluded that LG is superior to OG in terms of long-term survival and postoperative liver function recovery, and patients with cirrhosis experienced postoperative complications such as bleeding and wound infection (36). Zhu et al. showed that the surgical effect of LG is better than that of OG, and the involved patients developed ascites, wound infection, and other complications (37). Many studies revealed that LG is better than OG by comparing the efficacy and safety of LG and OG.

The surgical complications of gastrectomy are ascites, wound infection, and postoperative bleeding. Compared with OG, LG has a shorter operation time, less surgical blood loss, and shorter hospital stay (35, 36). Therefore, both LG and OG can be employed for the treatment of gastric cancer patients complicated with CTP A and CTP B cirrhosis. The effect of LG is better than that of OG. However, due to the small number of samples of gastric cancer patients with CTP C cirrhosis, the therapeutic effect of gastrectomy warrants further explore.

### Lymph Node Dissection

Gastric cancer often metastasizes to the lymph nodes, and doctors perform LN dissection at the same time as gastrectomy for radical excision. The extent of gastrectomy with lymph node dissection has been widely debated. According to the extent of lymph node dissection, LN dissection can be divided into D1 and D2 LN dissection. D1 LN dissection refers to the removal of perigastric lymph nodes, and D2 LN dissection refers to the removal of perigastric lymph nodes plus lymph nodes around the left stomach, common hepatic and splenic arteries, and coeliac axis (32).

Iwabu et al. reported that a 58-year-old man with gastric cancer and CTP B cirrhosis had a good prognosis without serious complications after undergoing gastrectomy with D1 LN dissection (38). Several multiple retrospective analyses confirmed that D2 LN dissection is safe for patients with gastric cancer and CTP A cirrhosis, and some patients in the study developed complications of ascites and wound infection (39, 40). Jang et al. demonstrated the feasibility of receiving D2 LN dissection in gastric cancer patients with CTP A cirrhosis by retrospective analysis. For patients with moderate or severe liver dysfunction, D1 or smaller ranges of LN dissection appears to be a more reasonable surgical option. The frequency of complications in patients with CTP B and C cirrhosis is higher than that of patients with CTP A cirrhosis. The involved complications mainly include ascites, wound infection, and hepatic encephalopathy (41). Guo et al. suggested that gastric cancer patients with CTP A cirrhosis could receive D1 or D2 LN dissection, while patients with CTP B cirrhosis could only receive D1 LN dissection. Moreover, it is very dangerous to perform LN dissection in patients with CTP C cirrhosis. In this study, 34 patients (58.6%) developed complications of ascites, postoperative bleeding, anastomosis leakage, pneumonia, and hepatic failure (43).

Complications of ascites, postoperative bleeding, and wound infection may occur in patients during LN dissection. How to determine the extent of LN dissection has been a difficulty in treatment, mainly because excessive LN dissection could lead to increased risk of postoperative bleeding and ascites, and small LN dissection may cause incurable treatment, affecting the prognosis of patients. The grade of liver cirrhosis should be considered when determining the extent of LN dissection. In general, gastric cancer patients with CTP A cirrhosis can receive D1 or D2 dissection, and patients with CTP B cirrhosis should receive D1 or less lymph node dissection. There is currently no good dissection treatment for CTP C cirrhosis patients. However, Lee et al. proposed that it might be feasible to improve the cirrhosis and then perform LN dissection for patients with CTP C cirrhosis (40).

### Other Treatment Methods

In addition to the treatments mentioned above for gastric cancer patients with cirrhosis, there are other cases reported with different treatment methods. Nishimura et al. reported that a 64-year-old woman with gastric cancer and CTP C cirrhosis first received liver transplantation, which improved liver and coagulation function. Nineteen days after liver transplantation, she underwent gastrectomy with no significant postoperative complications. This case provides new ideas for the treatment of gastric cancer patients with CTP C cirrhosis (44). A 68-year-old woman with high risk of advanced gastric cancer (AGC) and cirrhosis was reported to successfully receive laparoscopic and endoscopic cooperative surgery (LECS) without short-term postoperative complications nor recurrence after 6 months of follow-up. Therefore, LECS is a feasible palliative treatment for patients with AGC complicated with cirrhosis (45).

**Figure 1:**
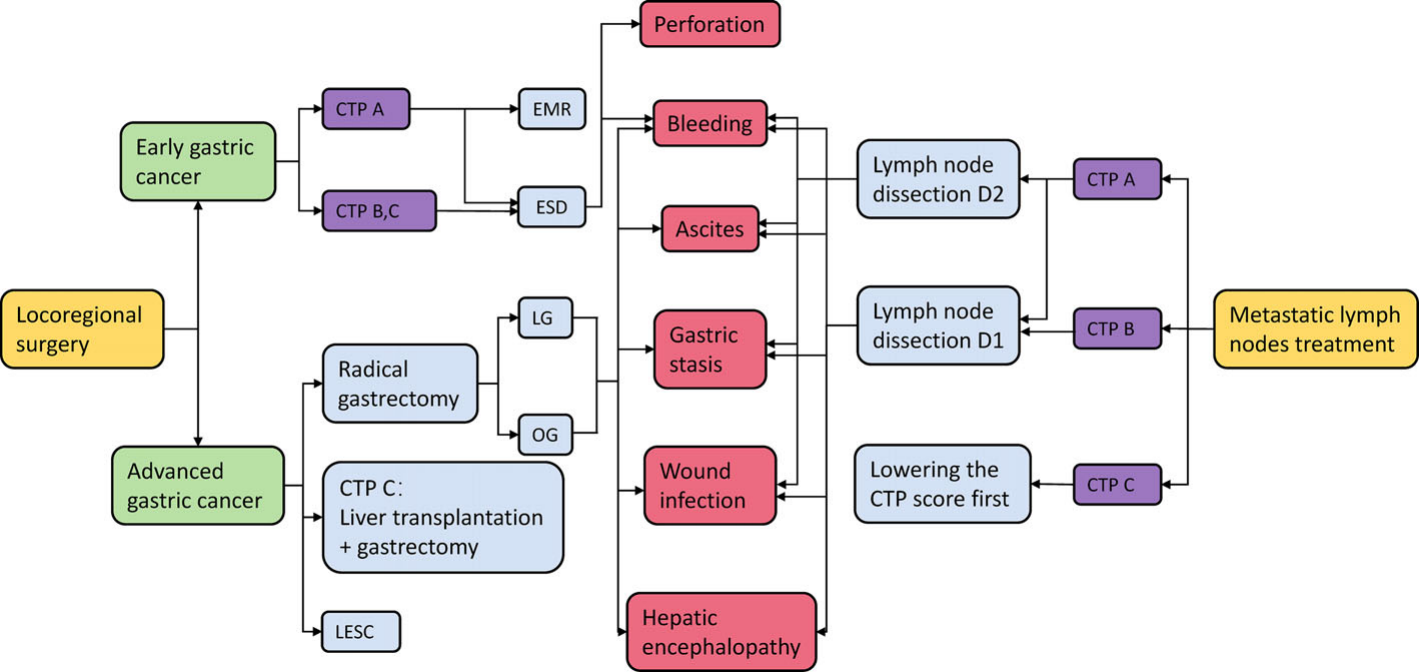
Gastric cancer treatment options and their possible complications. CTP A/B/C, Child–Turcotte–Pugh score A/B/C; EMR, endoscopic mucosal resection; ESD, endoscopic submucosal dissection; LG, laparoscopic gastrectomy; OG, ipen gastrectomy; LECS, laparoscopic and endoscopic cooperative surgery.

The surgical effects and prognosis of patients with EGC and AGC are different. The cirrhosis grade and postoperative complications also have a great influence on the treatment effect of patients. In the treatment of patients with gastric cancer and cirrhosis, cancer stage, cirrhosis grade, and postoperative complications should be taken into comprehensive consideration, so as to select the appropriate surgical plan for patients (**Figure 1**).

## Treatment of Esophageal Cancer With Cirrhosis and Its Influencing Factors

As the sixth most common cancer in the world, esophageal cancer is estimated to cause 450,000 deaths per year (46). There are two subtypes of esophageal carcinoma: squamous esophageal cell carcinoma and esophageal gland cancer. Since increased alcohol consumption is a common risk factor of esophageal cancer and cirrhosis, patients diagnosed with esophageal cancer and cirrhosis are not rare (47, 48). By analyzing the factors that affect postoperative mortality after esophagectomy, Sanz et al. found that cirrhosis is significantly associated with postoperative complications and mortality (49). Similarly, González-González et al. suggested that the presence of cirrhosis increases the incidence of complications (50). The presence of cirrhosis has a great impact on the surgery and prognosis of patients with esophageal cancer, so it is necessary to discuss the treatment methods and prognosis of esophageal cancer patients complicated with cirrhosis (**Table 2**).

**Table 2:** Summary of esophageal cancer stage, cirrhosis grade, surgical choice and prognosis.

Authors	Cancer stage	Cirrhosis grade	Management	Prognosis
**Shimakawa et al. (** 48 **)**	EEC	CTP B (n = 1)	Esophagectomy	The patient had no complications.
**Valmasoni et al. (** 49 **)**	EECAEC	CTP A (n = 71)CTP B (n = 2)	Esophagectomy	More respiratory events, infections and anastomotic complications occurred in patients.
**Wang et al. (** 50 **)**	EECAEC	CTP A (n = 30)CTP B (n = 7)	Esophagectomy	The rates of surgical death and postoperative hydrothorax were higher in cirrhosis patients.
**Lu et al. (** 51 **)**	–	CTP A (n = 10)CTP B (n = 4)CTP C (n = 2)	Esophagectomy	Five patients experienced 11 major complications.
**Tachibana et al. (** 52 **)**	EECAEC	CTP A (n = 11)CTP B (n = 7)	Esophagectomy	Operative death occurred in three patients.
**Cheng et al. (** 53 **)**	EECAEC	CTP A (n = 51)CTP B/C (n = 8)Unknown (n = 7)	Chemoradiotherapy Esophagectomy	Patients developed postoperative pneumonia, pleural effusion and chylothorax and had longer intensive care unit stay.
**Endlicher et al. (** 54 **)**	EEC	CTP B (n = 1)	EMR	The patient had recurrence 3 months after the surgery.
**Ciocîrlan et al. (** 55 **)**	EEC	CTP A/B (n = 4)	EMR	One patient died of mesenteric infarction 1 week after EMR
**Sawaguchi et al. (** 56 **)**	–	CTP A (n = 5)CTP B (n = 1)CTP C (n = 1)	ESD	The patients were treated successfully endoscopically. No adverse events occurred.
**Katano et al. (** 57 **)**	EEC	CTP B (n = 1)	ChemoradiotherapyESD	The patient had no recurrence in the 30 months since his treatment.
**Hagiwara et al. (** 58 **)**	AEC	CTP A (n = 1)	ChemoradiotherapyESD	The patient survived for more than 18 months after the initial treatment.
**Nishimura et al. (** 59 **)**	AEC	Unknown (n = 1)	Radiotherapy	The patient had no recurrence 30 months after the treatment.
**Maruyama et al (** 60 **)**	AEC	Unknown (n = 1)	Radiotherapy	The patient showed no recurrence eight months after the treatment.
**Trivin et al (** 61 **)**	–	CTP A (n = 22)CTP B (n = 4)	Radiochemotherapy	CTP A patients have better tolerance and prognosis than CTP B patients.

EEC, Early esophageal cancer; AEC, Advanced esophageal cancer; CTP A/B/C, Child–Turcotte–Pugh score A/B/C; EMR, endoscopic mucosal resection; ESD, endoscopic submucosal dissection.

### Esophagectomy

Esophagectomy is a treatment for resectable early esophageal cancer (EEC). A 52-year-old man with esophageal cancer and with alcoholic CTP B cirrhosis was reported to successfully receive esophagectomy with a good prognosis and no significant complications (62). Valmasoni et al. demonstrated that esophagectomy can be performed in esophageal cancer patients with cirrhosis in a cohort study (63). A retrospective study from China also confirmed that esophagectomy is a feasible and beneficial treatment option for patients with esophageal cancer and cirrhosis (51).

The cirrhosis grade can affect the safety and postoperative complications of esophagectomy. Schizas et al. confirmed that compared with patients with CTP B cirrhosis, there is a significant reduction in mortality from esophagectomy in those with CTP A cirrhosis (52). In evaluating the effectiveness of esophagectomy in patients with cirrhosis and non-cirrhosis, Deng et al. found that the incidence of pulmonary complications, pleural effusion, and anastomosis leakage in patients with cirrhosis is higher than that in patients without cirrhosis. Moreover, they proposed that patients with CTP A cirrhosis could receive esophagectomy, while patients with CTP B and C cirrhosis were unsuitable for esophagectomy because of the high risk of complications (53). This conclusion is consistent with that of Lu et al. (64). A retrospective analysis from Japan suggested that patients with CTP A or CTP B cirrhosis could be treated with esophagectomy despite the high morbidity and mortality. Postoperative complications in patients mainly include pleural effusion, recurrent laryngeal nerve paralysis (RLNP), and pneumonia (54). A retrospective study from China reported that esophageal cancer patients with CTP A cirrhosis can receive esophagectomy for treatment. Patients are prone to postoperative complications of pneumonia, pleural effusion, and chylothorax (55).

Generally, esophagectomy can be performed in esophageal cancer patients with CTP A cirrhosis, but it is not clear whether patients with CTP B and C cirrhosis can receive esophagectomy. Therefore, preoperative stratification and prevention of postoperative complications can effectively reduce the risk of surgery.

### EMR and ESD

Both endoscopic mucosal resection and endoscopic submucosal dissection are effective endoscopic resection methods. Endoscopic resection has a similar cure rate in some specialized centers compared to esophagectomy (56). Endlicher et al. reported that a 71-year-old woman was diagnosed with esophageal squamous cell carcinoma and alcoholic cirrhosis, and she successfully underwent EMR after ligation of esophageal varices without major bleeding complications (57). Ciocîrlan et al. reported four patients with early esophageal squamous cell carcinoma and cirrhosis, and they believed that EMR was feasible for their treatment (58). By evaluating the effectiveness and safety of ESD in the treatment of superficial esophageal carcinoma with cirrhosis and esophageal varices, a study from Japan concluded that ESD is a feasible modality for the treatment of esophageal squamous cell carcinoma in patients with cirrhosis (59).

Treatments of esophageal cancer with cirrhosis are not limited to single endoscopic resection. Katano et al. reported a case of esophageal submucosal invasive carcinoma that could not be surgically treated due to CTP B cirrhosis. They successfully treated patients with salvage ESD after chemoradiotherapy, and the patient had no obvious complications after surgery (60). A patient with advanced esophageal cancer (AEC) and CTP A alcoholic cirrhosis was reported to successfully undergo ESD after chemotherapy, and the patient had no significant postoperative complications (61).

According to the above studies, patients with cirrhosis complicated with esophageal cancer can accept ESD and EMR treatment with no obvious postoperative complications. However, the influence of cirrhosis grade on the effect of EMR and ESD surgery is not clear, and further study is needed.

### Chemoradiotherapy

Nishimura et al. reported a case of AEC complicated with cirrhosis in a 69-year-old man who was successfully treated with radiotherapy. The patient did not relapse within 30 months of treatment, which suggested that radiotherapy is an effective treatment for AGC with poor general condition (65). Moreover, a male patient with multiple superficial esophageal cancer and cirrhosis was reported to be successfully treated with radiotherapy, and he had no recurrence within 8 months (66). A retrospective study from France showed that patients with CTP A cirrhosis are tolerant to chemoradiotherapy, while patients with CTP B cirrhosis should be treated with weaker rather than conventional chemoradiotherapy regimens (67).

Radiotherapy is an effective way to treat esophageal cancer complicated with cirrhosis. The strategy of chemoradiotherapy should be based on the grade of cirrhosis of patients, and doctors should formulate an appropriate chemotherapy regime according to the general condition of patients.

**Figure 2:**
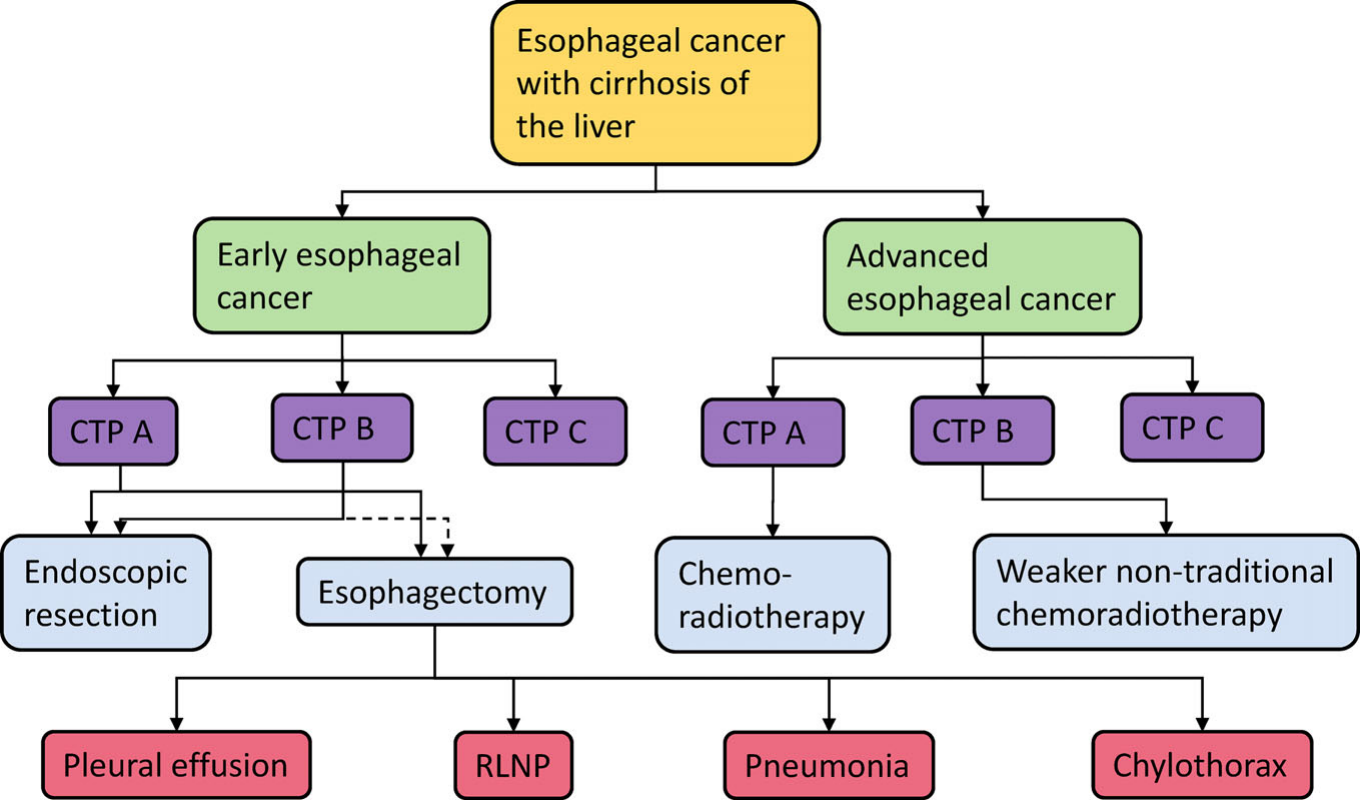
Esophageal cancer treatment options and their possible complications (The dotted line indicates inconsistent conclusions about whether patients can accept this treatment method). CTP A/B/C, Child–Turcotte-Pugh score A/B/C; RLNP, recurrent laryngeal nerve palsy.

Due to the lack of studies, the influence of cirrhosis grade on the treatment choice of esophageal cancer patients is not completely clear, and more efforts are needed to explore the relationship. When choosing an appropriate surgical plan for patients with esophageal cancer and cirrhosis, the stage of esophageal cancer, grade of cirrhosis, and postoperative complications should be comprehensively considered (**Figure 2**).

## Treatment of Colorectal Cancer With Cirrhosis and Its Influencing Factors

CRC is the third most common cancer worldwide and the second leading cause of cancer-related death (68). Most colorectal cancers are adenocarcinomas, and a few are squamous epithelial carcinoma and mucinous carcinoma. LC is a known risk factor of CRC (69, 70). CRC patients with cirrhosis are more likely to have complications than patients without cirrhosis. A cohort study from Danish population showed that the 30-day postoperative mortality was 24.1% in patients with cirrhosis and only 8.7% in patients without cirrhosis, indicating a significant increase in postoperative mortality (71). This is consistent with the results of other studies (72–76). Han et al. demonstrated that CRC patients with cirrhosis had a higher rate of intraoperative blood loss, higher likelihood of postoperative complications, and longer hospital stay (75). Shin et al. found that LC determines the prognosis of patients with CRC, regardless of cancer stage (73). CRC is a common disease, and surgery is its main treatment. LC is unfavorable for CRC surgery (73, 76).

Preoperative assessment of liver function is required to determine its severity before patients with CRC and cirrhosis undergo treatment, which could be evaluated by CTP score or MELD score (77, 78). By comparing the surgical outcomes of CRC patients with different cirrhosis grades, previous studies found that patients with CTP B cirrhosis had a higher incidence of complications and needed more intervention and longer hospital stay (76, 79). Meunier et al. reported that the leakage rate of CRC surgery in patients with cirrhosis was 18%, and nearly 60% of the patients suffered from CTP B or C cirrhosis (80). Severe cirrhosis of the liver, such as CTP B and C cirrhosis, may prolong wound healing and increase the risk of anastomosis leakage (81). The MELD score is also a good prognostic model for patients with cirrhosis (82, 83). CRC patients with cirrhosis could receive surgical treatment when the MELD score is <10. However, therapy to improve liver function should be performed until the MELD score is <10 when the MELD score is >10, in order to achieve better survival outcomes (75).

### Radical Resection

Radical resection aims to eradicate the primary tumor, such as partial and total removal of the colon and rectum, which can be further divided into laparotomy and open radical resection. In a retrospective study from China, the average blood loss in the CRC patients with cirrhosis who underwent open radical resection was high. These patients showed high recurrence and mortality rates in follow-ups (76). Bleeding during surgery is a common and worrying complication of cirrhosis (73, 84). It is reported that estimated blood loss in patients with cirrhosis who have undergone CRC surgery is between 148 and 245 ml, which is higher than normal surgical blood loss (75, 84). Due to increased bleeding caused by cirrhosis, the difficulty of performing open radical resection and the postoperative morbidity and mortality increase significantly.

Laparotomy has high feasibility, safety, and effectiveness in managing CRC patients with cirrhosis. Compared with open radical resection, laparotomy has great advantages in reducing blood loss, shortening hospital stay, and reducing complications (85, 86). Zhou et al. suggested that laparotomy can reduce postoperative complications in patients with CRC and cirrhosis to a certain extent. In some patients with cirrhosis, laparotomy appears to be a safe, less invasive surgical alternative that can reduce bleeding and improve early recovery without additional harm to patients (81).

Interestingly, there are some controversial conclusions regarding the treatment of CRC patients with LC. Montomoli et al. examined risk factors for 30-day mortality after surgery in patients with CRC complicated with cirrhosis. They found that the relative risk of laparotomy was 6.82, while that of open radical resection was 3.01, indicating that laparoscopic resection may have a higher risk of mortality than laparotomy (71). In addition, Sabbagh et al. suggested that open radical resection should be preferred in patients with cirrhosis for colon surgery (87).

### Adjuvant Therapy

Anticancer drugs must be carefully selected when chemoradiotherapy is performed on CRC patients with cirrhosis (88). In particular, the anticancer drug oxaliplatin can lead to increased risk of varicose veins, digestive hemorrhage, ascites, and portal hypertension in CRC patients, leading to poor prognosis of patients (89). Portal hypertension caused by cirrhosis must be taken into account. Madbouly et al. showed that chemoradiotherapy based on oxaliplatin does not significantly reduce cancer-specific mortality and may increase overall mortality and morbidity (90).

The use of vitamin K and the administration of fresh-frozen plasma for coagulation are alternative ideas for treatment. Careful control of bleeding may reduce postoperative bleeding (80, 91).

## Treatment of Pancreatic Cancer With Cirrhosis and Its Influencing Factors

The presence of cirrhosis can affect the outcomes of pancreatic cancer treatment and the risk of postoperative complications. In a study to verify the safety of pancreatic surgery in patients with cirrhosis, Warnick et al. demonstrated that cirrhosis increases the risk of postoperative complications in patients undergoing the surgery (92). Therefore, it is very important to investigate the treatment methods and postoperative complications of patients with pancreatic cancer combined with cirrhosis.

Pancreaticoduodenectomy is the most common treatment for pancreatic cancer. Sahaab et al. reported a 71-year-old patient with pancreatic cancer and cirrhosis, and he had a good prognosis after pancreaticoduodenectomy with minor chyle leak (93). A retrospective study from Egypt confirmed an increased risk of postoperative complications in patients with cirrhosis who underwent pancreaticoduodenectomy. There was a significant increase in wound complications, internal organ bleeding, pancreatic fistula, and hospital mortality in patients with cirrhosis (94). Schizas et al. proposed that wound infection, ascites, and anastomosis leakage are the most common postoperative complications in patients with cirrhosis who undergo pancreatic cancer surgery, while non-cirrhosis patients are less likely to develop these complications (95). It is of great significance to consider postoperative complications in the treatment of patients with pancreatic cancer and cirrhosis.

The cirrhosis grade has an impact on treatment options. Fuks et al. reported successful pancreaticoduodenectomy in four patients with CTP A cirrhosis, and they confirmed that pancreaticoduodenectomy is feasible in patients with CTP A cirrhosis and pancreatic cancer. Similarly, a retrospective study from France also suggested that pancreaticoduodenectomy is feasible for patients with CTP A cirrhosis, whereas CTP B cirrhosis remains a contraindication for pancreaticoduodenectomy (96). Therefore, patients with CTP A cirrhosis and pancreatic cancer can receive pancreaticoduodenectomy, and doctors should take a conservative attitude towards patients with CTP B and C cirrhosis for pancreaticoduodenectomy.

At present, pancreaticoduodenectomy is the main method for the treatment of pancreatic cancer complicated with LC. In the course of treatment, doctors should consider the grade of cirrhosis and the risk of postoperative complications in patients.

## Conclusion and Perspectives

Gastrointestinal cancers mainly include gastric cancer, esophageal cancer, colorectal cancer, and pancreatic cancer. Compared with non-cirrhosis patients with gastrointestinal cancer, patients with cirrhosis have poor treatment outcomes and prognosis. The grade of cirrhosis might limit the treatment choice of patients. Patients with mild cirrhosis can usually receive surgical treatment, while patients with severe cirrhosis should be treated conservatively. The consideration of postoperative complications plays an important role in choosing treatment modality in patients with gastrointestinal cancer and cirrhosis. Patients with gastrointestinal cancer and cirrhosis have an increased risk of postoperative complications after surgery. Different treatment methods may cause different postoperative complications, and the severity of postoperative complications is also different. More studies are needed to investigate the proper treatment options for patients with gastrointestinal cancer and different grade of LC.

In conclusion, the treatment of gastrointestinal cancer complicated with cirrhosis is not limited to the radical treatment of cancer. Doctors should consider the actual situation of gastrointestinal cancer, cirrhosis grade, and possible postoperative complications before treating patients.

## Author Contributions

ZX, YL, and CZ conceived and designed the article. TH and XH performed the literature search and data analysis. HZ and DJ drafted and critically revised the work. All authors contributed to the article and approved the submitted version.

## Funding

This study was supported by Beijing Bethune Charitable Foundation (No. B19296ES).

## Conflict of Interest

The authors declare that the research was conducted in the absence of any commercial or financial relationships that could be construed as a potential conflict of interest.

The reviewer XS declared a shared parent affiliation with several of the authors ZX, YL, CZ, TH, and XH to the handling editor at the time of the review.

## Publisher’s Note

All claims expressed in this article are solely those of the authors and do not necessarily represent those of their affiliated organizations, or those of the publisher, the editors and the reviewers. Any product that may be evaluated in this article, or claim that may be made by its manufacturer, is not guaranteed or endorsed by the publisher.

## Abbreviations

LC, liver cirrhosis; CTP, Child–Turcotte–Pugh; MELD, Model For End-Stage Liver Disease; INR, international normalized ratio; EMR, endoscopic mucosal resection; ESD, endoscopic submucosal dissection; EGC, early gastric cancer; LG, laparoscopic gastrectomy; OG, open gastrectomy; LN, lymph node; AGC, advanced gastric cancer; LECS, laparoscopic and endoscopic cooperative surgery; EEC, early esophageal cancer; RLNP, recurrent laryngeal nerve palsy; AEC, advanced esophageal cancer; CRC, colorectal cancer.
